# A Novel Antioxidant Multitarget Iron Chelator M30 Protects Hepatocytes against Ethanol-Induced Injury

**DOI:** 10.1155/2015/607271

**Published:** 2015-02-04

**Authors:** Jia Xiao, Yi Lv, Bin Lin, George L. Tipoe, Moussa B. H. Youdim, Feiyue Xing, Yingxia Liu

**Affiliations:** ^1^National Key Disciplines for Infectious Diseases, Shenzhen Third People's Hospital, Shenzhen 518112, China; ^2^Department of Immunobiology, Institute of Tissue Transplantation and Immunology, Jinan University, Guangzhou 510632, China; ^3^Department of Anatomy, The University of Hong Kong, Pokfulam, Hong Kong; ^4^Eve Topf Centers of Excellence, Technion, Rappaport Family Faculty of Medicine and Department of Pharmacology, 31096 Haifa, Israel

## Abstract

The multitarget iron chelator, M30, is a novel antioxidant and protective agent against oxidative stress in a spectrum of diseases. However, there is no report regarding its role in liver diseases. Since oxidative stress is one of the major pathological events during the progression of alcoholic liver diseases, the protective effects and mechanisms of M30 on ethanol-induced hepatocyte injury were investigated in this study. Rat hepatocyte line BRL-3A was pretreated with M30 prior to ethanol treatment. Cell death, apoptosis, oxidative stress, and inflammation were examined. Specific antagonists and agonists were applied to determine the involvements of hypoxia inducible factor-1 alpha (HIF-1*α*) and its upstream adenylate cyclase (AC)/cyclic AMP (cAMP)/protein kinase A (PKA)/HIF-1*α*/NOD-like receptor 3 (NLRP3) inflammasome pathway. We found that M30 significantly attenuated ethanol-induced cellular death, apoptosis, production of reactive oxygen species (ROS), and secretion of inflammatory cytokines and inhibited activation of the AC/cAMP/PKA/HIF-1*α*/NLRP3 inflammasome pathway. Inhibition and activation of the AC/cAMP/PKA/HIF-1*α* pathway mimicked and abolished the effects of M30, respectively. In conclusion, inhibition of the AC/cAMP/PKA/HIF-1*α*/NLRP3 inflammasome pathway by M30 partially contributes to its attenuation of hepatocyte injury caused by ethanol exposure.

## 1. Introduction

Alcoholic liver diseases (ALDs), including acute alcoholic liver injury, liver failure, alcoholic fatty liver disease (AFLD), and alcoholic steatohepatitis (ASH), annually result in an estimated 2.5 million deaths (4% of all mortality) worldwide [1]. Alcoholism or alcohol use disorder is defined as overconsumption of ethanol (men > 30 g/day and women > 20 g/day) [2]. The only definitive clinical treatment for ALD is liver transplantation. Abstinence is critical but usually cannot reverse advanced ALD. It should be accompanied by supportive therapy and nutritional management [3]. To date, although a spectrum of key signaling pathways and therapeutic targets have been described in ALD, the interaction between oxidative stress and inflammation is considered to be a central event during the initiation and progression of ALD [4, 5].

Inflammasomes are a group of large caspase-1-activating protein complexes in response to the evocation of innate immunity and the production of proinflammatory cytokines, interleukin-1*β* (IL-1*β*), and IL-18. Inflammasome activation has been shown to induce cell pyroptosis, a process of programmed cell death distinct from apoptosis [6]. Inflammasomes, particularly NOD-like receptor 3 (NLRP3) inflammasome, are shown to be activated in a variety of acute and chronic liver diseases, including ALD [7]. Our previous study found that* Lycium barbarum* polysaccharide attenuated ethanol-induced hepatocyte injury partially through regulating the thioredoxin-interacting protein- (TXNIP-) NLRP3 inflammasome pathway [7]. However, the upstream regulators of NLRP3 during ALD progression await further investigation.

Hypoxia-inducible factor-1 alpha (HIF-1*α*) is activated by hypoxia and is a master regulator of cellular redox status and downstream production of proinflammatory cytokines (e.g., tumor necrosis factor-alpha (TNF-*α*) and IL-6) [8]. It could be activated by increased cellular oxygen levels, primarily at the protein level [9]. Recent studies pointed out that under hypoxic or oxidative stress environments, adenylate cyclase (AC) activates HIF-1*α* through cyclic AMP (cAMP)/protein kinase A (PKA) pathway [10]. Since ethanol induces hypoxia and elevates HIF-1*α* in the liver [11], it was speculated that HIF-1*α* might be a novel therapeutic target for ethanol-induced injury and an upstream regulator of NLRP3 inflammasome.

In the current study, we aimed to determine whether the AC/cAMP/PKA/HIF-1*α* pathway and NLRP3 inflammasome are involved in the protective effect of a novel multitarget iron chelator (M30) against ethanol-induced hepatocyte injury* in vitro*.

## 2. Materials and Methods

### 2.1. Drugs and Chemicals

The multifunctional iron chelator, M30 (5-[N-methyl-N-propargylaminomethyl]-8-hydroxyquinoline) was synthesized and kindly provided by Varinel Inc. (Philadelphia, PA) [12, 13]. CAY10585 (HIF-1*α* inhibitor) was purchased from Cayman Chemical (Ann Arbor, MI). Forskolin (AC agonist), SQ22536 (AC inhibitor), H89 (PKA inhibitor), and db-cAMP (stable cAMP analogue) were purchased from Sigma-Aldrich (St. Louis, MO). Antibodies against catalase (CAT), glutathione peroxidase 1 (GPx1), NLRP3, apoptosis-associated speck-like protein containing a CARD (ASC), caspase-1, and *β*-actin were ordered from Abcam (Cambridge, UK).

### 2.2. Cell Culture and Treatments

Rat normal hepatocyte cell line BRL-3A was supplied by the Cell Bank of Type Culture Collection of Chinese Academy of Sciences (Shanghai, China). Cells were cultured in DMEM with 10% (v/v) FBS at 37°C with 5% CO_2_. Before drug treatment, cells must reach a confluence of 60–70%. For the pretreatment with M30, PBS dissolved M30 was added 2 hours before the ethanol treatment. For the treatments using agonists and inhibitors of the AC/cAMP/PKA/HIF-1*α* pathway, drugs were added along with M30 or individually 2 hours before the ethanol treatment at designated concentrations.

### 2.3. MTT Assay

The cell viability was evaluated by the 3-(4,5-dimethylthiazol-2-yl)-2,5-diphenyltetrazolium bromide (MTT, Sigma-Aldrich, St. Louis, MO) method. After drug treatment, cells were washed by sterile PBS for 3 times and then incubated with 5 mg/mL MTT for 3 hours and subsequently dissolved in dimethyl sulfoxide (DMSO). The absorbance of cells was measured at 570 nm. The percent of cell viability was defined as the relative absorbance of treated cells versus untreated cells.

### 2.4. Detection of Alanine Aminotransferase (ALT) Levels

After treatments, cell supernatant was collected and the level of ALT was detected by an ALT/GPT kit (EIAab Science, Wuhan, China) according to manufacturer's instruction.

### 2.5. Quantification of Apoptotic Cells

After treatment, Hoechst 33342 (Sigma, 5 *μ*g/mL) and propidium iodide (Sigma, 5 *μ*g/mL) were added to each well to stain live cells. The cell population was separated into 3 groups: live cells showed only a low level of fluorescence; apoptotic cells showed a higher level of blue fluorescence; and dead cells showed low-blue and high-red fluorescence. Stained cells were observed and quantified by two independent cell biologists without knowing the grouping. The results were expressed as the percentage of apoptosis (PA): PA = apoptotic cell number/total cell number × 100% [14].

### 2.6. Caspase-3/7 Activity

The activity of caspase-3/7 in cell lysate was measured using the Cell Meter Caspase-3/7 Activity Apoptosis Assay Kit (AAT Bio., Sunnyvale, CA) according to the user manual. The results were read at 520 nm in a microplate reader (Bio-Rad) and expressed as fold change of the control.

### 2.7. Measurement of ROS Production

Intracellular production of ROS was detected by a fluorescence probe—2′,7′-dichlorofluorescin diacetate (DCFH-DA, Sigma-Aldrich), as previously described [7]. Briefly, after treatment, cells were washed three times with PBS and then incubated with 10 *μ*M DCFH-DA for 30 min at 37°C for green fluorescent light visualization. Quantification of green fluorescence was analyzed by using ImageJ software (Version 1.48, National Institutes of Health, Bethesda, MD). Fluorescent quantification results were then normalized by the protein amount of each specific plate well (~110–150 *μ*g/well of a 24-well plate).

### 2.8. GSH/GSSG Ratio Measurement

To determine the intracellular oxidative status, the ratio of reduced glutathione (GSH) to oxidized glutathione (GSSG) in each cellular protein sample was measured by using a GSH/GSSG detection assay kit from Abcam (Cambridge, UK).

### 2.9. RNA Extraction and Quantitative PCR

Total cellular RNA was extracted using illustra RNAspin mini kit (GE healthcare, UK). The preparation of the first-strand cDNA was conducted following the instruction of the SuperScript First-Strand Synthesis System (Invitrogen, Carlsbad, CA). The mRNA expression levels of target genes were measured by Takara SYBR premix Taq quantitative PCR system (Takara Bio Inc., Shiga, Japan) and in MyiQ2 real-time PCR machine (Bio-Rad, Hercules, CA). Parallel amplification of glyceraldehyde-3-phosphate dehydrogenase (GAPDH) was used as an internal control. Relative quantification was calculated using the 2^−ΔΔCt^ method. Primers and PCR conditions were described in previous literature [15]. The relative expression of the specific gene to the internal control was obtained and then expressed as percentage of the control value. All real-time PCR procedures including the design of primers, validation of PCR environment, and quantification methods were performed following the MIQE guideline [16].

### 2.10. Western Blot

Western blot analyses of cell lysates (cytosolic or nuclear fractions) were performed as previously described [17]. The ratio of the optical density of the protein product to the internal control (*β*-actin) was obtained and the data were expressed as ratio or percentage of the control.

### 2.11. Enzyme-Linked Immunosorbent Assay (ELISA) Assay

The levels of secreted TNF-*α*, IL-1*β*, and IL-6 in culture medium were measured using corresponding ELISA kits from PeproTech (PeproTech Inc., Rocky Hill, NJ) according to user instructions. ELISA assay kit for secreted IL-18 was purchased from Invitrogen (Carlsbad, CA). The level of HIF-1*α* was measured using an EIISA kit from Abcam (Cambridge, UK).

### 2.12. Statistical Analysis

Data were expressed as means ± SEM. Comparison between groups was examined using the Kruskal-Wallis test followed by Dunn's post hoc test. A value of *P* < 0.05 was considered to be statistically significant (Prism 5.0, Graphpad software, Inc., San Diego, CA).

## 3. Results

### 3.1. M30 Inhibited Ethanol-Induced Hepatocyte Injury and Apoptosis

**Figure 1:**
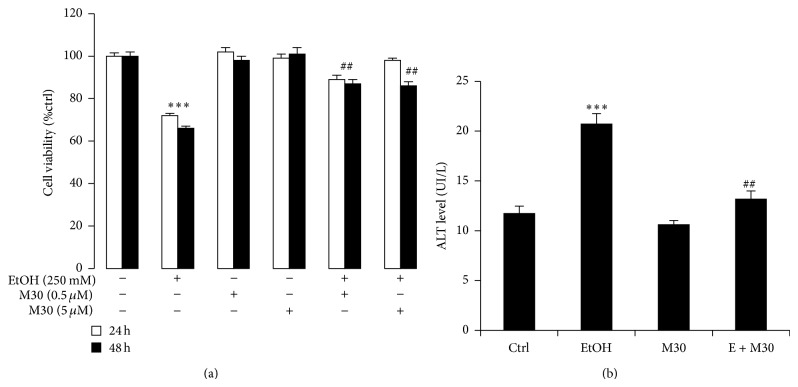
M30 improved hepatocytes injury. (a) Cell viability after 24-hour and 48-hour treatments and (b) released ALT concentration in the culture medium. Data from each group (*n* = 4) were expressed as means ± SEM. Statistical comparison between groups was done using the Kruskal-Wallis test followed by Dunn's post hoc test to detect differences in all groups. “∗∗∗” means significantly different from control group (*P* < 0.001). “##” means significantly different from ethanol exposure group (*P* < 0.01). “###” means significantly different from ethanol exposure group (*P* < 0.001). EtOH, ethanol, M30, vehicle M30 treatment, E + M30, ethanol + M30 cotreatment.

Based on previous literatures [18, 19], we selected 0.5 *μ*M and 5 *μ*M M30 in the current study. Cell viability assay suggested that, after 24-hour exposure to ethanol, around 30% of BRL-3A cells became inviable. M30 pretreatment at both concentrations significantly increased the cell viability (Figure 1(a)). A 48-hour treatment with ethanol and/or M30 exhibited similar results, indicating that the protective effects of M30 on ethanol-damaged hepatocyte mainly occurred within the first 24 hours (Figure 1(a)). Therefore, we selected 0.5 *μ*M and 24 hours as the incubation concentration and duration for the following studies. In line with the cell viability results, pretreatment with 0.5 *μ*M M30 significantly reduced ethanol exposure caused release of ALT from damaged hepatocyte into the culture medium (Figure 1(b)).

**Figure 2:**
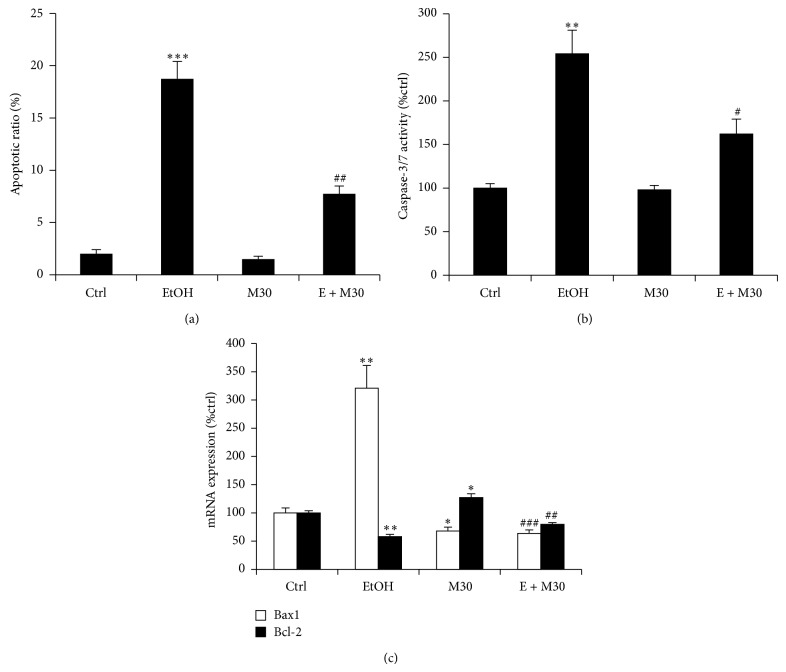
M30 improved cellular apoptosis. (a) Apoptotic ratio after various treatments. (b) Activity of caspase-3/7 of hepatocytes after various treatments. (c) Cellular Bcl-2 and Bax1 mRNA change after various treatments. Data from each group (*n* = 4) were expressed as means ± SEM. Statistical comparison between groups was done using the Kruskal-Wallis test followed by Dunn's post hoc test to detect differences in all groups. “∗” means significantly different from control group (*P* < 0.05). “∗∗” means significantly different from control group (*P* < 0.01). “∗∗∗” means significantly different from control group (*P* < 0.001). “#” means significantly different from ethanol exposure group (*P* < 0.05). “##” means significantly different from ethanol exposure group (*P* < 0.01). “###” means significantly different from ethanol exposure group (*P* < 0.001). EtOH, ethanol, M30, vehicle M30 treatment, E + M30, ethanol + M30 cotreatment.

To examine the effects of M30 on cellular apoptosis, we firstly measured the apoptotic ratio of BRL-3A after various treatments. We found that ethanol exposure greatly increased apoptosis of the cell, from ~2% to ~18% (~9-fold) and pretreatment of M30 partially abolished this effect of ethanol (Figure 2(a)). Quantification of cellular caspase-3/7 activity, antiapoptotic gene Bcl-2, and proapoptotic gene Bax1 further supported these observations (Figures 2(b) and 2(c)).

### 3.2. M30 Inhibited Ethanol-Induced Hepatocyte Oxidative Stress

**Figure 3:**
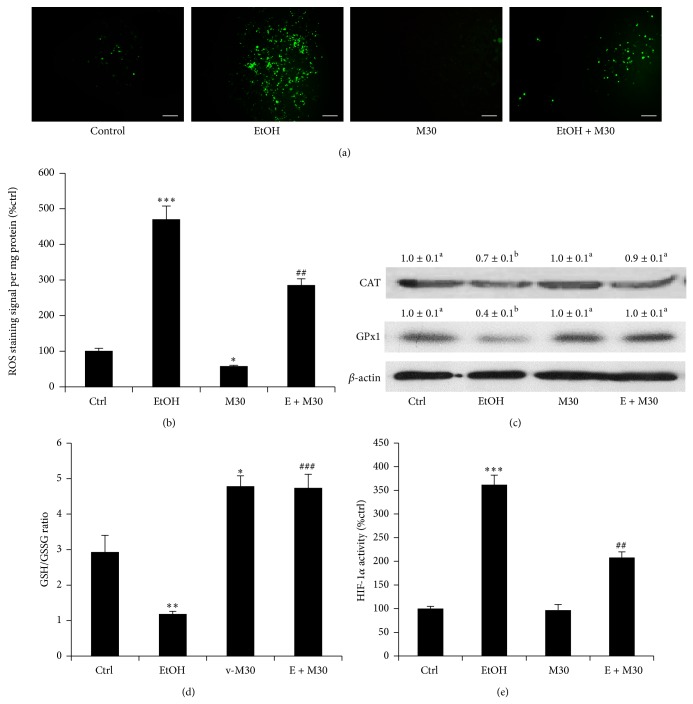
M30 improved cellular oxidative stress. (a) Production of ROS staining in the cells after various treatments (magnification 200x, bar = 100 *μ*m). (b) Quantification of ROS staining. (c) Expressional changes of antioxidants CAT and GPx1 after various treatments. (d) Change of GSH/GSSG ratio treatments. (e) Changes of transcriptional activity of HIF-1*α* in each group. Data from each group (*n* = 4) were expressed as means ± SEM. Statistical comparison between groups was done using the Kruskal-Wallis test followed by Dunn's post hoc test to detect differences in all groups. “∗” means significantly different from control group (*P* < 0.05). “∗∗” means significantly different from control group (*P* < 0.01). “∗∗∗” means significantly different from control group (*P* < 0.001). “##” means significantly different from ethanol exposure group (*P* < 0.01). “###” means significantly different from ethanol exposure group (*P* < 0.001). Letters “a” labelled above the values of control, M30, and ethanol + M30 groups mean there is no significant change among these groups (*P* > 0.05). Letter “b” means the value of ethanol-treated group is statistically different from other three values (*P* < 0.05). EtOH, ethanol, M30, vehicle M30 treatment, E + M30, ethanol + M30 cotreatment.

Since formation of ROS and oxidative stress are main consequences of ethanol metabolism in the hepatocytes, we then tested the production of ROS in the culture medium and within the hepatocyte after ethanol and/or M30 incubation. Fluorescence staining results suggested that ethanol obviously increased the signal of ROS staining, while M30 reduced it (Figures 3(a) and 3(b)). This phenomenon was consistent with the finding that ethanol exposure significantly reduced the protein expression level of antioxidant enzymes CAT and GPx1, which was recovered by the pretreatment with M30 (Figure 3(c)). In addition, the status change of cellular oxidative stress was exhibited by the ratio change of cellular GSH/GSSG, since this ratio is an indicator of cellular health, with reduced GSH constituting up to 98% of cellular GSH under normal conditions [20]. Ethanol treatment significantly reduced the GSH/GSSG ratio from ~2.9 to ~1.2. Pretreatment with M30, either independently or in combination with ethanol, significantly increased the ratio to a level (~4.7) even higher than that of the control group, strongly suggesting the antioxidant property of M30 (Figure 3(d)). We also quantified the activity of HIF-1*α*, one of the major regulators of oxidative stress generation in alcoholic liver diseases [9]. In agreement with other studies, ethanol significantly induced the activity of HIF-1*α*, which was reversed by the pretreatment with M30. Vehicle-M30 treatment did not influence the basal activity level of HIF-1*α* (Figure 3(e)).

### 3.3. M30 Attenuated Ethanol-Induced Hepatocyte Inflammatory Responses and NLRP3 Inflammasome Activation

**Figure 4:**
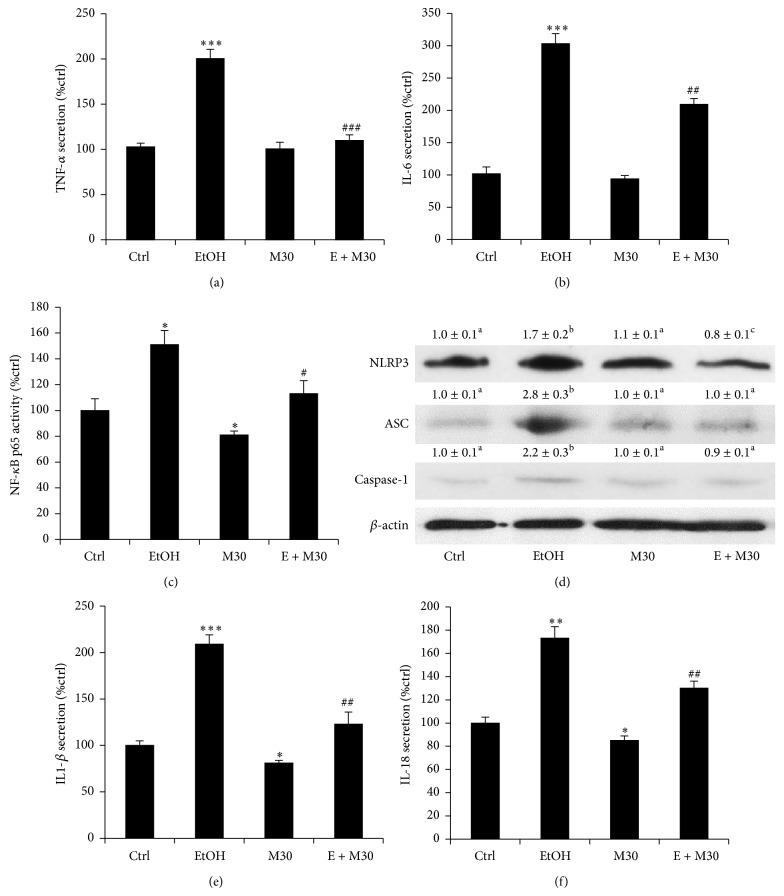
M30 improved cellular inflammation and NLRP3 inflammasome activation. (a, b) Changes of secreted levels of TNF-*α* and IL-6 in each group after treatments. (c) Change of transcriptional activity of NF-*κ*B p65 subunit after treatments. (d) Expressional changes of NLRP3, ASC, and caspase-1 proteins after treatments. (e, f) Changes of secreted levels of IL-1*β* and IL-18 in each group after treatments. Data from each group (*n* = 4) were expressed as means ± SEM. Statistical comparison between groups was done using the Kruskal-Wallis test followed by Dunn's post hoc test to detect differences in all groups. “∗” means significantly different from control group (*P* < 0.05). “∗∗” means significantly different from control group (*P* < 0.01). “∗∗∗” means significantly different from control group (*P* < 0.001). “#” means significantly different from ethanol exposure group (*P* < 0.05). “##” means significantly different from ethanol exposure group (*P* < 0.01). “###” means significantly different from ethanol exposure group (*P* < 0.001). Letters “a” labelled above the values of control, M30, and ethanol + M30 groups mean there is no significant change among these groups (*P* > 0.05). Letter “b” means the value of ethanol-treated group is statistically different from other three values (*P* < 0.05). For NLRP3 protein, letter “c” on ethanol + M30 group means significant change when compared with other three groups (i.e., control, ethanol, and M30, *P* < 0.05). EtOH, ethanol, M30, vehicle M30 treatment, E + M30, ethanol + M30 cotreatment.

Overproduction of ROS and oxidative stress in the liver after ethanol exposure often induce potent inflammatory responses, which may significantly aggravate the pathogenesis of ALD [21]. Thus, whether pretreatment with M30 attenuates ethanol-induced proinflammatory cytokines production and NLRP3 inflammasome activation is another important aim for the current study. ELISA results of secreted TNF-*α* and IL-6 showed that ethanol incubation greatly induced the secretion of these two proinflammatory cytokines from BRL-3A cells, indicating an inflammatory status of the cells. M30 totally or partially abolished such effects (Figures 4(a) and 4(b)). This phenomenon was further confirmed by the change of transcription activity of NF-*κ*B, the master regulator of both oxidative stress and inflammation in the liver (Figure 4(c)). To characterize the involvement of NLRP3 inflammasome in ethanol-induced hepatocyte damage and M30-mediated protection, the cellular protein levels of NLRP3, ASC, and caspase-1, as well as the secreted protein levels of both IL-1*β* and IL-18, were measured by Western blot or ELISA in all groups. Ethanol exposure significantly upregulated all the protein levels of these key components of NLRP3 inflammasome, indicating that NLRP3 inflammasome was activated during ethanol incubation. Pretreatment with M30 significantly reduced their levels (Figures 4(d)–4(f)).

### 3.4. Inhibition of HIF-1*α* Reduced NLRP3 Inflammasome Activation

**Figure 5:**
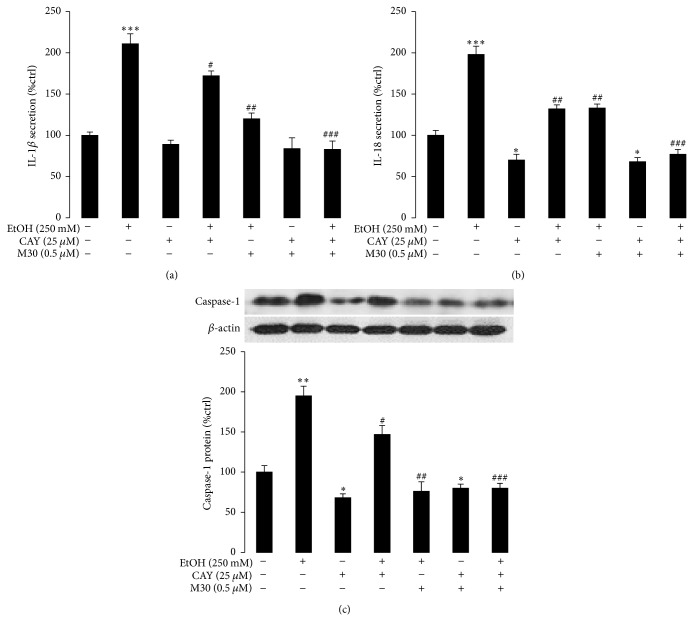
HIF-1*α* is critical for the NLRP3 inflammasome activation. (a–c) Changes of secreted IL-1*β*, IL-18, and cellular caspase-1 expressions after ethanol exposure, M30 pretreatment, and/or HIF-1*α* inhibitor CAY10585. Data from each group (*n* = 4) were expressed as means ± SEM. Statistical comparison between groups was done using the Kruskal-Wallis test followed by Dunn's post hoc test to detect differences in all groups. “∗” means significantly different from control group (*P* < 0.05). “∗∗” means significantly different from control group (*P* < 0.01). “∗∗∗” means significantly different from control group (*P* < 0.001). “#” means significantly different from ethanol exposure group (*P* < 0.05). “##” means significantly different from ethanol exposure group (*P* < 0.01). “###” means significantly different from ethanol exposure group (*P* < 0.001).

The production of IL-1*β* is a central step in ALD progression. Thus, delineating the upstream signals that lead to the activation of NLRP3 inflammasome and IL-1*β* secretion is important for the pathological study of this disease [22, 23]. Since recent several reports suggested that there were HIF-1*α* response elements in the human and mouse* Il1b* promoter [24], it was interesting to find whether blockade of HIF-1*α* could reduce the production of IL-1*β* and other inflammasome products (IL-18 and caspase-1) in our system. The HIF-1*α* inhibitor CAY10585 was able to significantly decrease ethanol induced IL-1*β* production without affecting its basal level (Figure 5(a)). Addition of M30 further reduced the production of IL-1*β* to a control-comparable level, indicating that HIF-1*α* was indeed a regulating target of M30, but there should be other targets that M30 used to play its protective roles against ethanol (Figure 5(a)). Change of IL-18 and caspase-1 protein level further confirmed this observation (Figures 5(b) and 5(c)).

### 3.5. M30 Modulates AC/cAMP/PKA Pathway and Its Downstream HIF-1*α*

**Figure 6:**
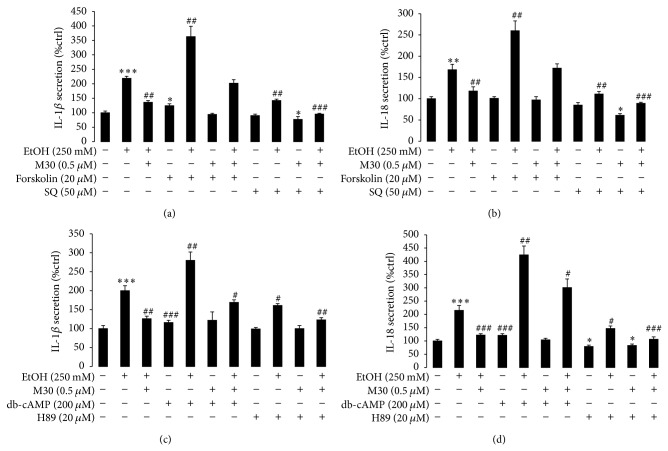
Role of the AC/cAMP/PKA pathway in NLRP3 inflammasome activation. (a-b) Changes of secreted IL-1*β* and IL-18 after ethanol exposure, M30 pretreatment, and/or AC agonist forskolin/inhibitor SQ22536. (c-d) Changes of secreted IL-1*β* and IL-18 after ethanol exposure, M30 pretreatment, and/or cAMP analogue db-cAMP/PKA inhibitor H89. Data from each group (*n* = 4) were expressed as means ± SEM. Statistical comparison between groups was done using the Kruskal-Wallis test followed by Dunn's post hoc test to detect differences in all groups. “∗” means significantly different from control group (*P* < 0.05). “∗∗” means significantly different from control group (*P* < 0.01). “∗∗∗” means significantly different from control group (*P* < 0.001). “#” means significantly different from ethanol exposure group (*P* < 0.05). “##” means significantly different from ethanol exposure group (*P* < 0.01). “###” means significantly different from ethanol exposure group (*P* < 0.001).

Since PKA activates HIF-1*α* through cAMP response element-binding protein (CREB) [25] and AC is reported to be the target of other hepatoprotective agents [26], we then considered that the AC/cAMP/PKA pathway might be repressed by M30 to downregulate the activity of HIF-1*α*. To test this hypothesis, AC activator forskolin and inhibitor SQ22536 were used to treat BRL-3A cells in the presence or absence of ethanol and M30. We found that vehicle forskolin slightly increased the basal secretion of IL-1*β* but not IL-18. When pretreated with M30 before ethanol, forskolin significantly reduced the effect of M30 on IL-1*β* and IL-18 secretion. Similarly, when AC was inhibited by SQ22536, the secretion of IL-1*β* and IL-18 induced by ethanol exposure was significantly decreased (Figures 6(a) and 6(b)). Pretreatment with SQ22536 before ethanol potentiated the effect of M30 on the reduction of IL-1*β* and IL-18 secretion. Collectively, these data suggested that M30 exerted its beneficial effects on hepatocytes partially through the action of AC.

We then used a stable analogue of cAMP (db-cAMP) and an inhibitor of PKA (H89) to see the change of IL-1*β* and IL-18 secretion in our system. In line with the results from AC overactivation and repression, db-cAMP significantly impaired the effects of M30 on both IL-1*β* and IL-18 secretion while H89 partially mimicked the effects of M30 (Figures 6(c) and 6(d)), indicating that the cAMP/PKA pathway was also applied by M30 when treating alcoholic hepatocyte injury.

## 4. Discussion

M30 is one of the most effective drugs possessing iron-chelating/radical scavenging potency and inhibition of lipid peroxidation features. Cytochrome P450 isoenzyme inhibition and voltage-dependent potassium channel blocking studies showed that M30 was not cytotoxic in several cell lines [12, 13]. M30 has been shown to inhibit oxidative stress and inflammation in several diseases, including type 2 diabetes mellitus [27], Alzheimer's disease [28], Parkinson's disease [29], and drug-induced brain cell injury [30]. However, little is known about its effect on the liver. In this study, we demonstrated that pretreatment with M30 significantly preserved cell viability, attenuated cell damage, and improved cellular oxidative stress and inflammation in an* in vitro *ethanol-induced hepatocyte injury model. Pharmacological activation and inhibition by specific agonist and inhibitors of signaling molecules further confirmed the partial involvement of the AC/cAMP/PKA/HIF-1*α* and the NLRP3 inflammasome pathways in the hepatoprotective actions of M30.

A number of previous papers reported that ethanol evoked HIF-1*α* activation in the liver. For example, ethanol treatment induced fatty liver was alleviated in mice with hepatocyte-specifically knockdown of HIF-1*α*, while overexpression of HIF-1*α* aggravated that damage [31]. Wang et al. also found that blocking HIF-1*α* activation and subsequent action had therapeutic implication against ethanol/CYP2E1-induced oxidative stress, steatosis, and liver injury [11]. In our study, after ethanol exposure, the activity of HIF-1*α* was significantly increased along with exacerbated hepatocyte damage, oxidative stress, inflammation, and NLRP3 inflammasome activation when compared with those in the control group. M30 pretreatment significantly reduced HIF-1*α* activity and other cellular injuries. Correspondingly, ethanol-induced NLRP3 inflammasome activation was decreased in cells treated with HIF-1*α* inhibitor CAY10585. M30 addition further reduced the inflammasome activation. Collectively, these data suggested that HIF-1*α* might play important roles in ethanol-induced hepatocyte injury and M30-mediated hepatoprotection. This conclusion was consistent with a very recent paper finding that moderate hypoxia potentiates NLRP3 inflammasome activation and IL-1*β* production in activated human macrophages [32]. Opposite to our findings, several previous studies suggested that M30 could ameliorate neuronal oxidative stress through the stabilization and induction of HIF-1*α* protein. This may be attributed to the different downstream signalings in different organs (brain versus liver) [27–29]. Indeed, further studies are warranted to delineate this discrepancy.

The direct link between the AC/cAMP/PKA pathway and the activation of HIF-1*α* has been recently examined in several cell models, such as epithelial-mesenchymal transition in lung cancer [33], pancreatic beta cell injury [34], and inflammation in macrophage [10]. We demonstrated that activation and inhibition of the AC/cAMP/PKA pathway could, respectively, increase and decrease the production of inflammasome products (IL-1*β* and IL-18). Indeed, the signaling mediator in the upstream of AC (particularly the membrane receptor) that immediately transduces ethanol-induced damage and M30-mediated protection is the key missing link for the current study. Based on the studies that adenosine 1A or 2A receptor antagonist prevented and reversed liver injury in mouse models of ALD [35, 36] and that adenosine 2A receptor directed the activation of NLRP3 inflammasome through the AC/cAMP/PKA/HIF-1*α* pathway [10], we highly speculated that adenosine receptors could be the key immediate molecules modulating the beneficial effects of M30. This hypothesis deserves future investigations.

In conclusion, pretreatment with multitarget iron chelator and antioxidant M30 attenuates ethanol-induced injury in rat BRL-3A hepatic cells. The hepatoprotective property of M30 is partially achieved by modulating the AC/cAMP/PKA/HIF-1*α* pathway. This study also provides a rationale for developing hepatoprotective therapies that target HIF-1*α* or its up/downstream mediators.

## Abbreviations

AC: Adenylate cyclase
AFLD: Alcoholic fatty liver disease
ALD: Alcoholic liver disease
ALT: Alanine aminotransferase
ASC: Apoptosis-associated speck-like protein containing a CARD
ASH: Alcoholic steatohepatitis
cAMP: Cyclic AMP
CAT: Catalase
DCFH-DA: 2′,7′-Dichlorofluorescin diacetate
DMSO: Dimethyl sulfoxide
ELISA: Enzyme-linked immunosorbent assay
GAPDH: Glyceraldehyde-3-phosphate dehydrogenase
GPx1: Glutathione peroxidase 1
GSH: Glutathione
GSSG: Oxidized glutathione
HIF-1*α*: Hypoxia inducible factor-1 alpha
IL: Interleukin
NLRP: NOD-like receptor protein
PKA: Protein kinase A
ROS: Reactive oxygen species
TNF: Tumor necrosis factor
TXNIP: Thioredoxin-interacting protein.
